# Supplementary material for: A Tonic Signaling Code Predicts CAR-T Cell Efficacy in Diffuse Midline Glioma
Source: bioRxiv. 2025 Oct 1:2025.09.29.679095. Preprint. [Version 1] doi: 10.1101/2025.09.29.679095 (PMC12621713; doi:10.1101/2025.09.29.679095)
Supplement: 1 [file NIHPP2025.09.29.679095v1-supplement-1.pdf]

bioRxiv preprint doi: https://doi.org/10.1101/2025.09.29.679095; this version posted October 1, 2025. The copyright holder for this preprint (which was not certified by peer review) is the author/funder, who has granted bioRxiv a license to display the preprint in perpetuity. It is made available under aCC-BY 4.0 International license.

Supplementary Figure Legends:

Figure S1. B7H3.BC CAR-T cells have better killing efficacy against DIPG cells in vitro than do MGA271 and Hu8H9 CAR-T cells.
(A) Flow cytometric analysis of B7-H3 expression on DIPG cells.
(B) Quantification of B7-H3 expression as measured by mean fluorescence intensity (MFI). Representative of three independent experiments.
(C) Representative fluorescence images of B7-H3 or control CAR-T cells in co-culture experiments with DIPG-C1 (left) and DIPG21 (right) cells. The CAR-T cells (green) were co-cultured with DIPG cells (red) at a ratio of 1:4 for DIPG-C1 and 1:2 for DIPG21. Images were captured on days 0, 1, 2, 3, and 5 or 6. Representative of three donors.
(D) MFIs of AF647-conjugated B7-H3 protein in cultures of indicated CAR-T cells. Representative of three independent experiments.

Figure S2. B7H3.BC CAR-T cells maintain a stem-like, less differentiated phenotype.
(A) Representative flow cytometry plots showing the differentiation states of control and B7-H3 CAR-T cells on day 14 of culture. Surface expression of CD62L and CD45RO defines the following populations: naïve T cells/stem cell memory T cells ($CD62L^{+}CD45RO^{-}$), central memory T cells ($CD62L^{+}CD45RO^{+}$), effector memory T cells ($CD62L^{-}CD45RO^{+}$), and effector T cells ($CD62L^{-}CD45RO^{-}$). Representative of three donors.
(B) Percentages of different populations in cultures of control and B7-H3 CAR-T cells on day 14 of culture.
(C) Percentages of naïve T cells/stem cell memory T cells (left) and effector memory T cells (right) in cultures of control and B7-H3 CAR-T cells on day 14 of culture.
(D) Representative flow cytometric analysis of stem-like markers (CD62L, CCR7, and CD45RA) and the memory marker (CD45RO) on CAR-T cells and control T cells after 14 days of culture. Representative of three donors.
(E) Quantification of marker expression based on mean fluorescence intensity (MFI) CAR-T cells and control T cells after 14 days of culture.
(F) Flow cytometric analysis of stemness markers CD62L, CCR7, and CD45RA and the memory marker CD45RO on CAR-T cells and control T cells after 7 days of culture. Representative of three donors.
(G) Quantification of marker expression in panel based on MFI.
For panels **C**, **E** and **G**, unpaired two-tailed Student's *t*-test. $^{**}P < 0.01$, $^{***}P < 0.001$.

Figure S3. Detection of CAR expression and Structural modeling of scFv dimers and binding affinity analysis.
(A) Western blot analysis for GFP and CAR CD3ζ in three B7-H3 CAR-T cells on day 10 of culture.
(B) Representative dimer structures of each scFv predicted by AlphaFold2-Multimer, selected based on the highest confidence scores. Individual monomers are shown in blue and green. Blue and green coloring identifies different monomers.
(C) The Rosetta energy score showing the binding affinity of three scFv dimers.
(D) Pulling work measured by steered molecular dynamics (SMD) simulations for dissociating scFv monomers. A total of 100 replicas (20 replicas for each of 5 structural models) were shown. Error bars represent 95% CI.
(E) Binding energy estimated using Jarzynski's Equality based on 20 independent replicas of the pulling simulations for each dimer structure. Error bars represent 95% CI.
C-E, unpaired one-tailed or two-tailed Student's t-test. $^{*}P < 0.05$, $^{**}P < 0.01$, $^{***}P < 0.001$.

bioRxiv preprint doi: https://doi.org/10.1101/2025.09.29.679095; this version posted October 1, 2025. The copyright holder for this preprint (which was not certified by peer review) is the author/funder, who has granted bioRxiv a license to display the preprint in perpetuity. It is made available under aCC-BY 4.0 International license.

Figure S4. B7H3.BC CAR-T cells have long-term antitumor activity in rechallenge assays.
(A-C) Representative flow cytometry plots of mCherry-labeled DIPG13 cells and GFP-labeled CAR-T cells after (**A**) one, (**B**) two, and (**C**) three rounds of co-culture. CAR-T cells and tumor cells were co-cultured at a ratio of 1:4. Representative of three donors.

Figure S5. B7H3.BC CAR-T cells exhibit high efficacy in spheroid models.
(A) Schematic of the co-culture experiment. Patient-derived DIPG cells were seeded in 8-well glass chambers to allow spheroid formation. After 3 days, CAR-T cells were added.
(B) Representative fluorescence images of B7H3.BC CAR-T cells in co-culture with patient-derived DIPG spheroids. Images were captured at indicated days post-CAR-T cell addition. Representative of three donors.
(C) Representative confocal images of B7H3.BC and MGA271 CAR-T cells in co-culture with DIPG36 3D-spheroids. Images were captured at indicated timepoints post-CAR-T cell addition.
(D) mCherry intensities at 18 hours. Representative of three donors.
D, unpaired two-tailed Student's t-test. *P < 0.05.

Figure S6. B7H3.BC CAR-T cells have high antitumor efficacy in vivo.
(A-C) A) Schematic overview of the experiment.
B) Bioluminescence images showing tumor progression (n = 8 per group).
C) Kaplan-Meier survival curve of NSG mice (n = 8 per group).

Figure S7. mRNA-based CAR-T cells demonstrate potent antitumor activity against DIPG.
(A) Representative fluorescence images of B7H3.BC mRNA-based CAR-T cells in co-culture with indicated DIPG cells. The ratio of T cells (green) to DIPG cells (red) was 1:1. Scale bars = 200 µm. Representative of three donors.
(B) Percentage survival of DIPG13 (upper), DIPG21 (middle) and DIPG36 (lower) at days 2 and 5 of incubation with indicated T cells. Data are means ± SEM.
C) Schematic of experiment.
D) Bioluminescence images of NSG mice.
E) DIPG36 tumor growth over time.
F) Kaplan-Meier survival curve.
G) H&E staining of brain sections. The areas highlighted in red boxes are shown at higher magnification below. The dotted outlines indicate the boundaries of visible tumor regions.
For panel **B**, *t*-test. ***P < 0.001. For panel **E**, unpaired Student's *t*-test. For panel **F**, the log-rank test was used. *P < 0.05.

Figure S8. Transcriptomic analyses reveal a stem-like, less exhausted phenotype in B7H3.BC CAR-T.
(A) Principal component analysis of RNA-seq data from untransduced T cells, control T cells (Ctrl), and B7H3.BC, MGA271 and Hu8H9 CAR-T cells on day 12 of culture.
(B) Heatmap of differentially expressed genes across untransduced T cells, control T cells, and B7H3.BC, MGA271 and Hu8H9 CAR-T cells on day 12 of culture.
(C) Gene Ontology analysis of upregulated pathways (red) and downregulated pathways (blue) in B7H3.BC CAR-T cells compared to MGA271 and Hu8H9 CAR-T cells.
(D) Metascape transcription factor analysis of genes downregulated in B7H3.BC CAR-T cells compared to MGA271 and Hu8H9 CAR-T cells.
(E) Heatmap of expression of selected genes associated with tonic signaling-related pathways.

bioRxiv preprint doi: https://doi.org/10.1101/2025.09.29.679095; this version posted October 1, 2025. The copyright holder for this preprint (which was not certified by peer review) is the author/funder, who has granted bioRxiv a license to display the preprint in perpetuity. It is made available under aCC-BY 4.0 International license.

(F) Pathway enrichment analysis of genes downregulated in B7H3.BC CAR-T cells compared to MGA271 and Hu8H9 CAR-T cells.
(G) Representative GSEA plots highlighting representative significantly downregulated pathways in B7H3.BC CAR-T cells compared to MGA271 or Hu8H9 CAR-T cells.
(H) Heatmap of expression of selected genes related to stemness, activation, differentiation, and exhaustion across groups.
(I) GSEA analysis showing differential enrichment of KLF2 target genes and the T cell exhaustion signature in B7H3.BC and MGA271 CAR-T cells.
(**J**) Heatmap of expression of genes involved in oxidative phosphorylation and glycolysis pathways in B7H3.BC, MGA271, and Hu8H9 CAR-T cells relative to control T cells.
(K) GSEA analysis highlighting representative metabolic pathways enriched in B7H3.BC CAR-T cells or in MGA271 and Hu8H9 CAR-T cells.
(L) GO network analyses of pathways significantly upregulated in B7H3.BC CAR-T cells relative to the MGA271 and Hu8H9 CAR-T cells, control T cells transduced with empty vector (Ctrl), and untransduced T cells.
(M) Heatmap of expression of genes in the cGAS-STING pathway across different groups.
(N) GSEA analysis of the cGAS-STING pathway between B7H3.BC CAR-T and the other groups.

Figure S9. Epigenomic analyses reveal a stem-like, less exhausted phenotype.
(A) Heatmap showing H3K27ac signal intensities near the TSS of genes downregulated in B7H3.BC CAR-T. The control T cells and CAR-T cells were cultured for 12 days after infection.
(B) Heatmap of H3K27ac signal intensities at promoters or enhancers of associated with T cell exhaustion and tonic signaling in CAR-T cells relative to control T cells.
(C, D) Representative tracks depicting H3K27ac enrichment at loci associated with **C)** T cell exhaustion, tonic signaling, and **D)** stemness-related genes in CAR-T cells.
(E) Representative tracks depicting H3K27ac enrichment at the loci of housekeeping genes.

Figure S10. Clustering analysis of RNA-seq data to derive distinct CAR-T cell scores.
(A) Clustering plots showing scaled expression changes of genes in CTR (untransduced and Ctrl), B7H3.BC CAR-T, and Other CAR-T (MGA271 and Hu8H9). Gene expression values were normalized to z-scores for visualization.
(B) Heatmaps depicting differentially expressed genes associated with CAR-T effector-memory (CAR-Tem) signature, and CAR-T inflammatory (CAR-Infl) signature as indicated.
(C) The CAR-Tem score and CAR-Infl score in untransduced T cells, control T cells, and B7H3.BC, MGA271 and Hu8H9 CAR-T cells. The scores were calculated by the ssGSEA method based on the corresponding gene lists.

Figure S11. Single-cell RNA-seq analysis reveals changes across control and B7-H3 CAR-T cells.
(**A**) Dot plot of marker gene expression in different T cell populations. Dot color represents the scaled average expression of marker genes in different cell populations. Dot size indicates the proportion of cells.
(B) Proportions of CD8 TSCM, CD8 TCM and CD4/CD8 Tpex-like cells in control T and CAR-T cells.
(C) Quantification of the proportion of $TCF1^{+}TIM3^{+}$ cells in B7-H3 CAR-T cells on day 13 of culture. Representative of three donors.
(D) Violin plot of CAR-Tem scores in control and B7-H3 CAR-T cells. The crossbar represents mean value of the scores.
C, unpaired two-tailed Student's t-test. **D**, Kruskal-Wallis test followed by pairwise Wilcoxon rank-sum tests (BH-adjusted). ***P < 0.001.

bioRxiv preprint doi: https://doi.org/10.1101/2025.09.29.679095; this version posted October 1, 2025. The copyright holder for this preprint (which was not certified by peer review) is the author/funder, who has granted bioRxiv a license to display the preprint in perpetuity. It is made available under aCC-BY 4.0 International license.

Figure S12. The correlation of CAR-T scores with clinical outcome.

(A-D) Comparison of T cell stemness scores (**A**), Tpex scores (**B**), CAR-Tem scores (**C**) or CAR-Infl scores (**D**) in CAR-T non-responders and responders using the datasets from *Deng et al., 2020* (non-responder, n = 14; responder, n = 9) (left) or *Haradhvala et al., 2022* (non-responder, n = 14; responder, n = 16) (right). The scores were calculated using the ssGSEA method.

(E) ROC curves with corresponding AUCs displaying the predictive performance of the CAR-Ton scores in distinguishing CAR-T non-responders from responders in the datasets from *Deng et al., 2020* (left) and *Haradhvala et al., 2022* (right), and calculated by the ssGSEA method.

(F) Heatmap of tonic signaling gene expression in CAR-T and IL-15 CAR-T patients using the data from Dataset (*Steffin et al., 2024*).

A-D, unpaired two-tailed Mann–Whitney test. ns, not significant.

bioRxiv preprint doi: https://doi.org/10.1101/2025.09.29.679095; this version posted October 1, 2025. The copyright holder for this preprint (which was not certified by peer review) is the author/funder, who has granted bioRxiv a license to display the preprint in perpetuity. It is made available under aCC-BY 4.0 International license.

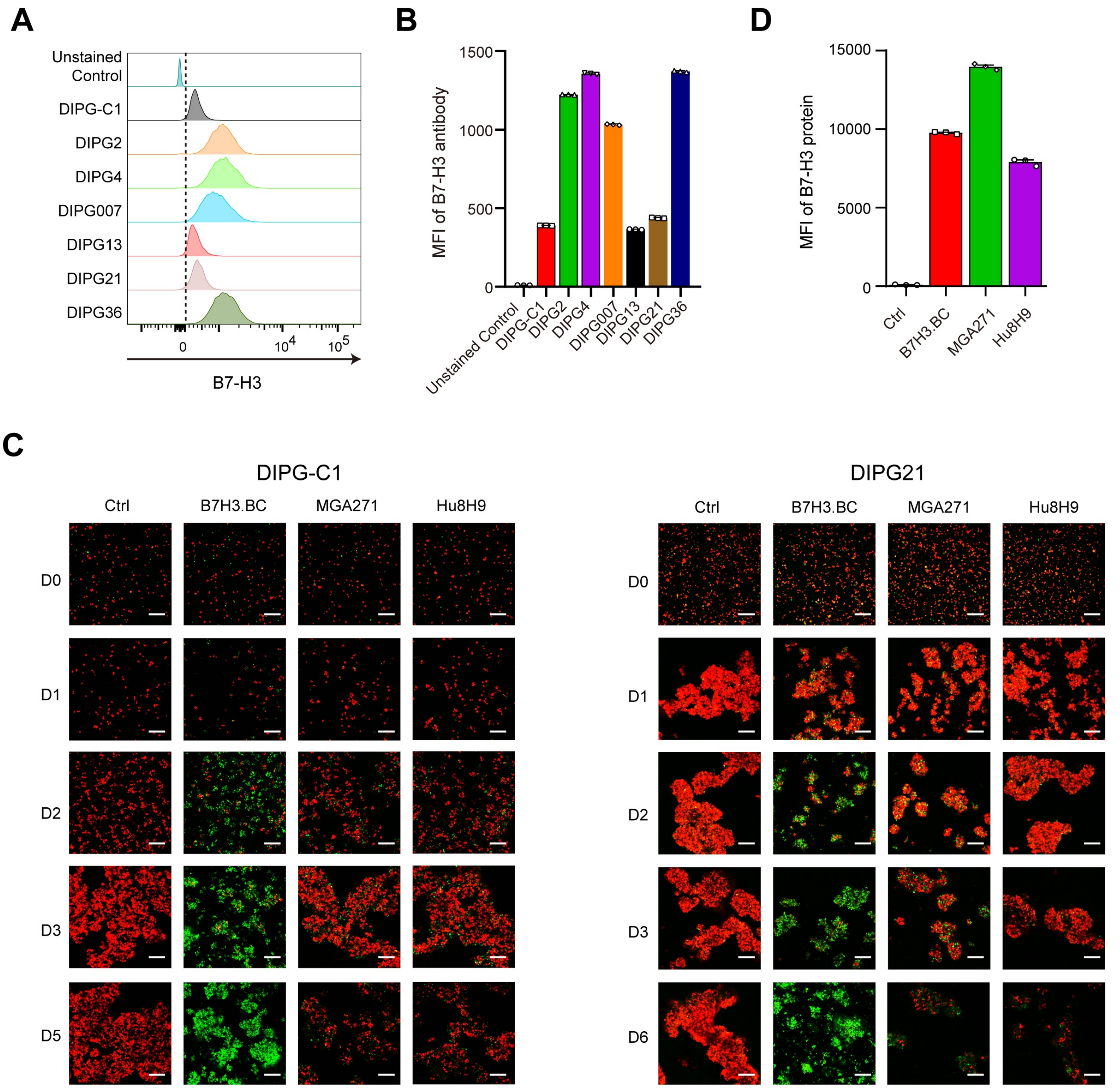

Supplementary Figure S1

bioRxiv preprint doi: https://doi.org/10.1101/2025.09.29.679095; this version posted October 1, 2025. The copyright holder for this preprint (which was not certified by peer review) is the author/funder, who has granted bioRxiv a license to display the preprint in perpetuity. It is made available under aCC-BY 4.0 International license.

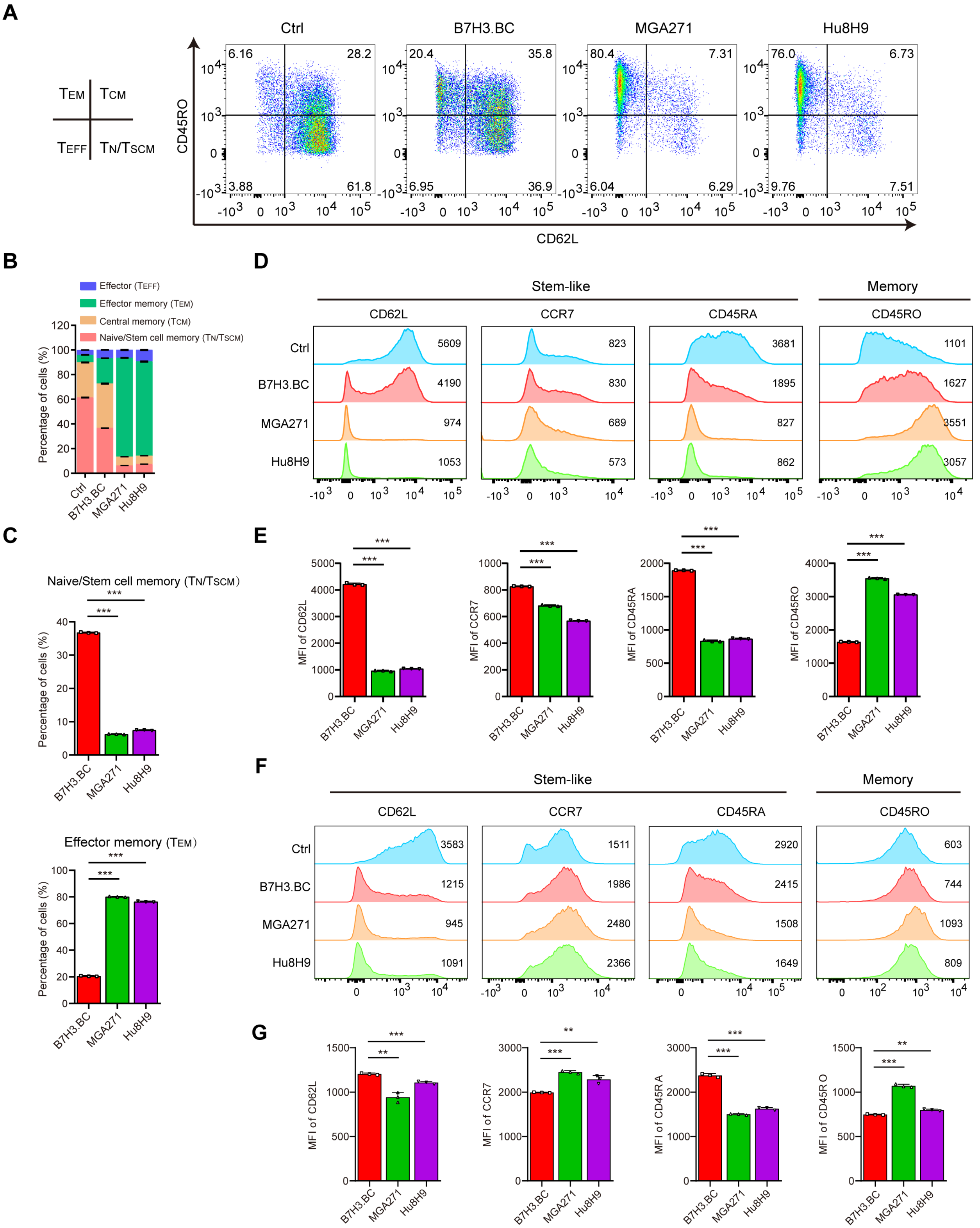

Supplementary Figure S2

bioRxiv preprint doi: https://doi.org/10.1101/2025.09.29.679095; this version posted October 1, 2025. The copyright holder for this preprint (which was not certified by peer review) is the author/funder, who has granted bioRxiv a license to display the preprint in perpetuity. It is made available under aCC-BY 4.0 International license.

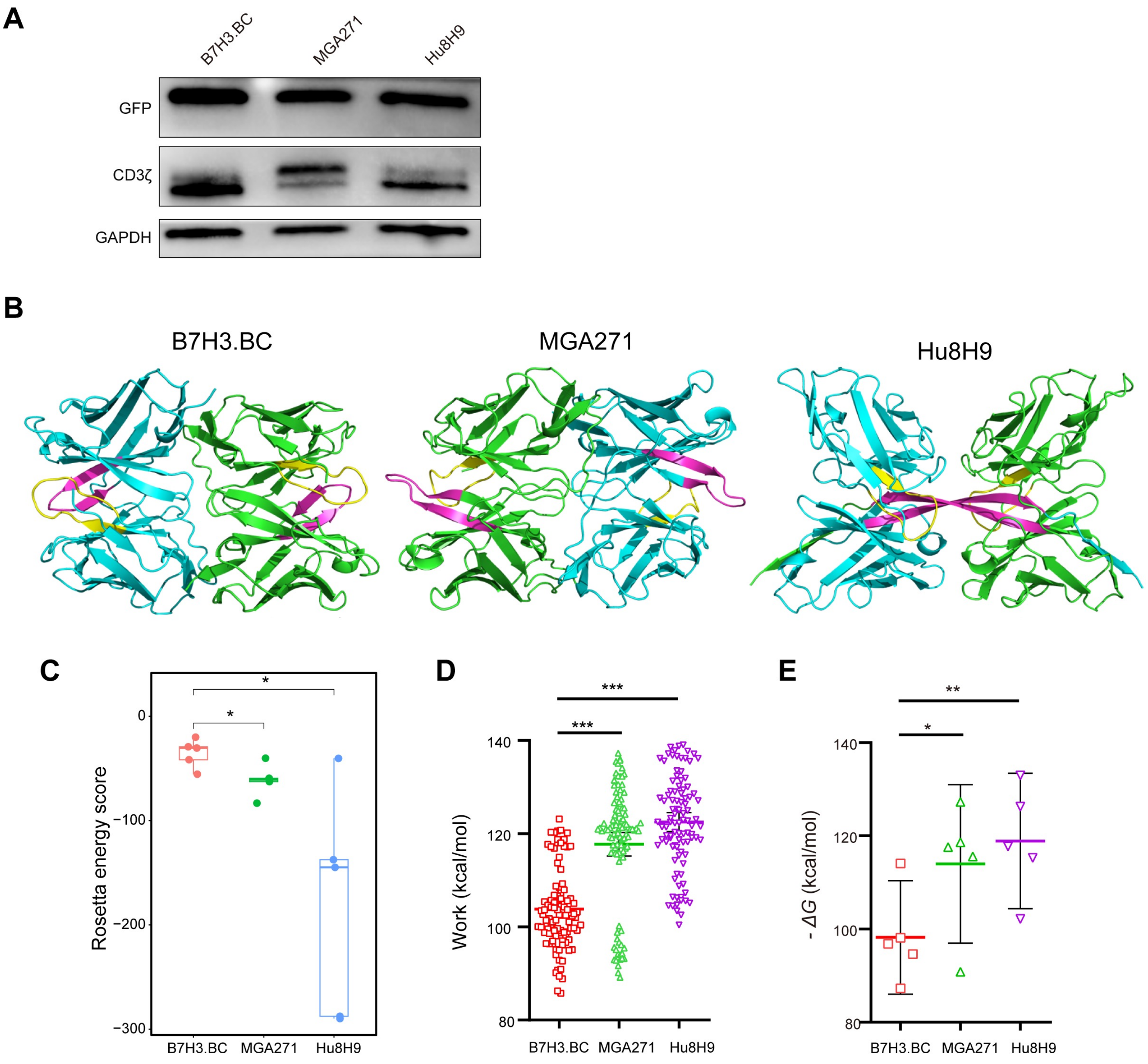

Supplementary Figure S3

bioRxiv preprint doi: https://doi.org/10.1101/2025.09.29.679095; this version posted October 1, 2025. The copyright holder for this preprint (which was not certified by peer review) is the author/funder, who has granted bioRxiv a license to display the preprint in perpetuity. It is made available under aCC-BY 4.0 International license.

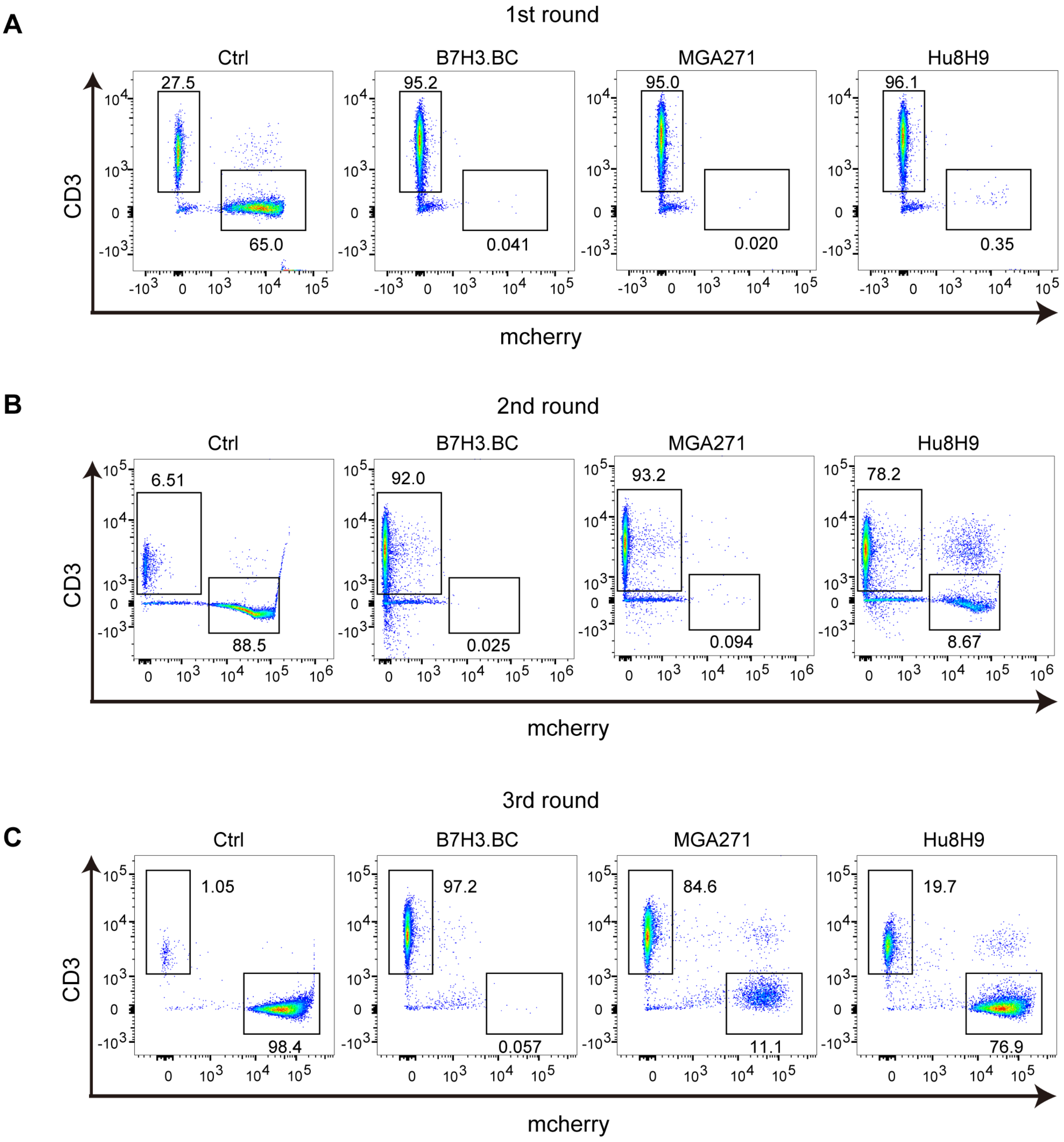

Supplementary Figure S4

bioRxiv preprint doi: https://doi.org/10.1101/2025.09.29.679095; this version posted October 1, 2025. The copyright holder for this preprint (which was not certified by peer review) is the author/funder, who has granted bioRxiv a license to display the preprint in perpetuity. It is made available under aCC-BY 4.0 International license.

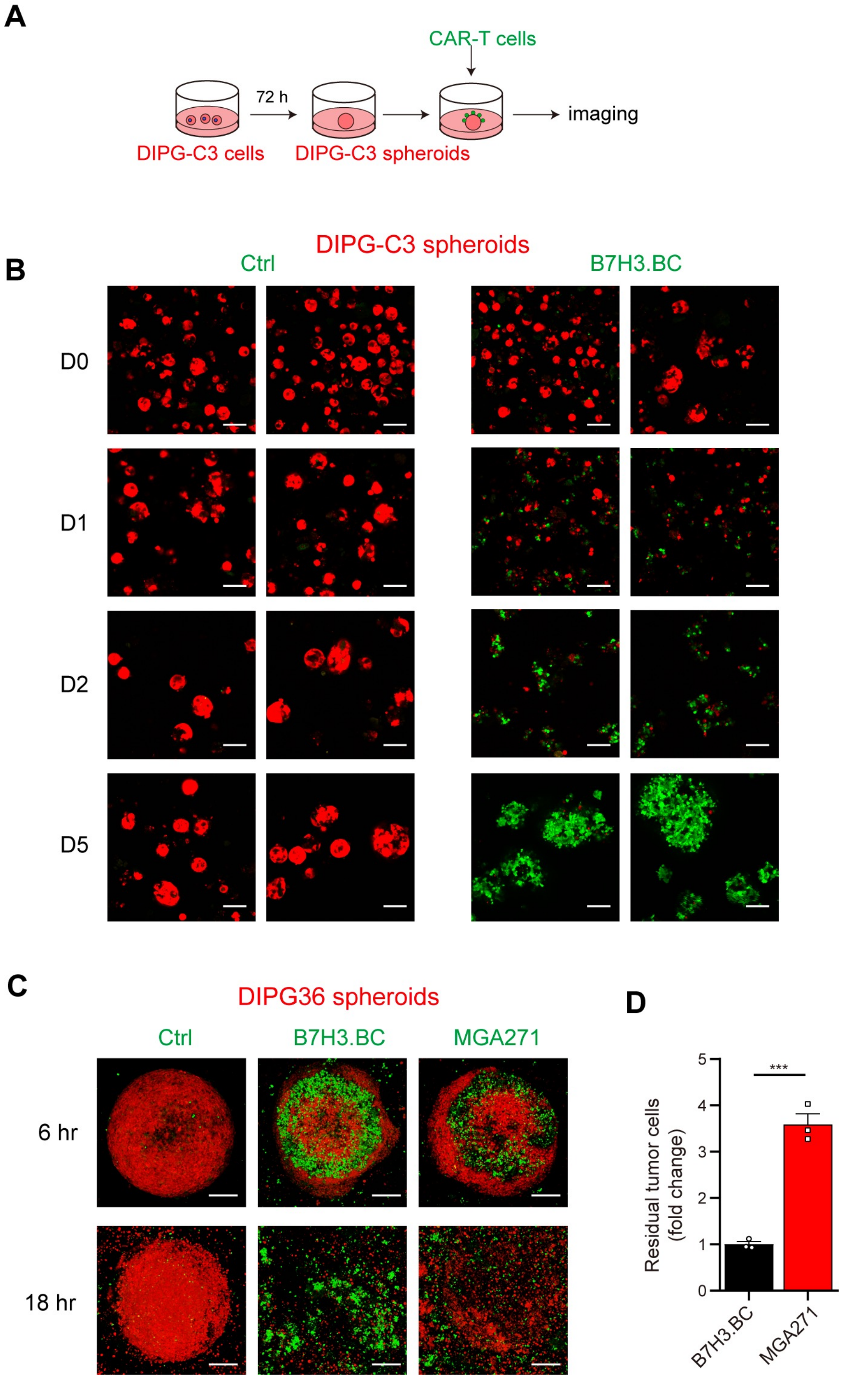

Supplementary Figure S5

bioRxiv preprint doi: https://doi.org/10.1101/2025.09.29.679095; this version posted October 1, 2025. The copyright holder for this preprint (which was not certified by peer review) is the author/funder, who has granted bioRxiv a license to display the preprint in perpetuity. It is made available under aCC-BY 4.0 International license.

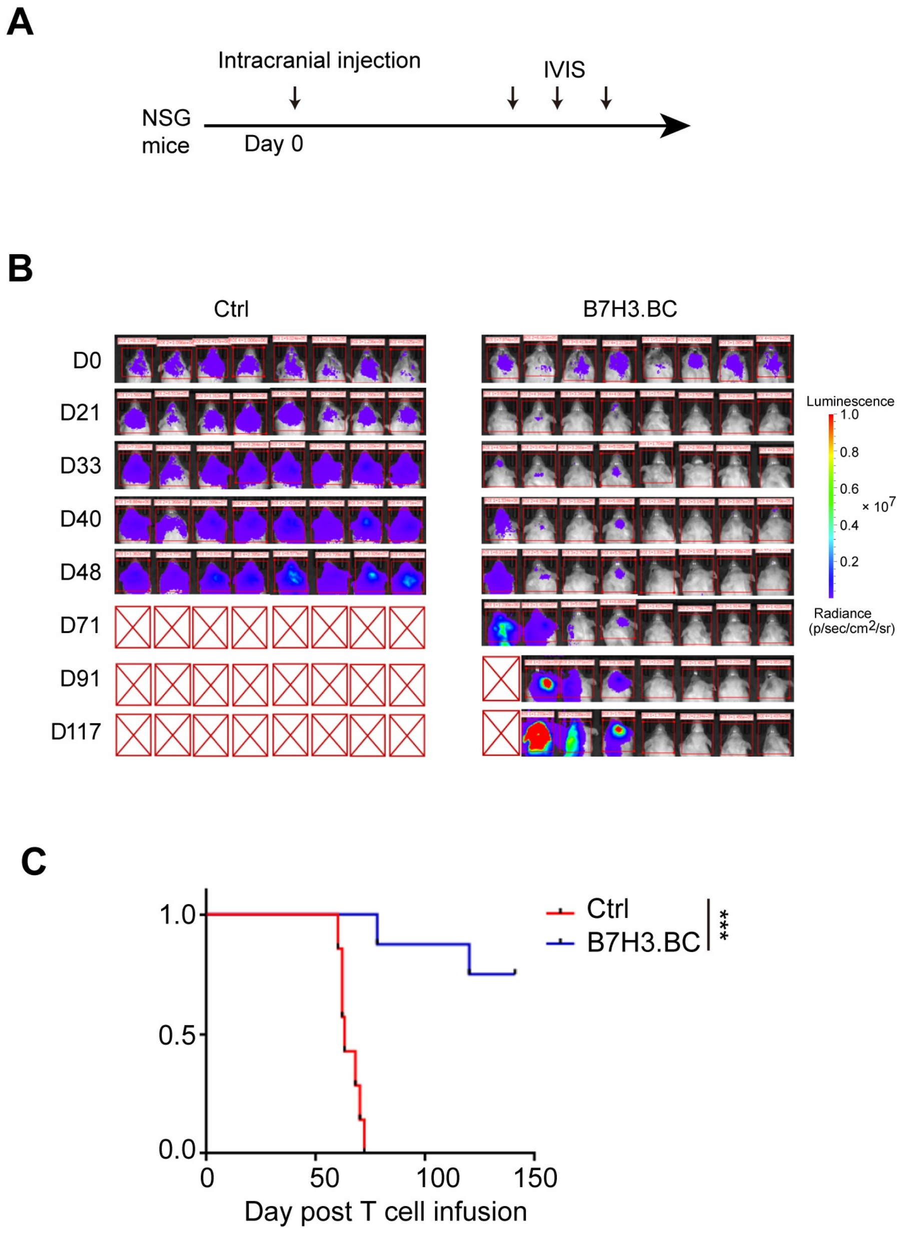

Supplementary Figure S6

bioRxiv preprint doi: https://doi.org/10.1101/2025.09.29.679095; this version posted October 1, 2025. The copyright holder for this preprint (which was not certified by peer review) is the author/funder, who has granted bioRxiv a license to display the preprint in perpetuity. It is made available under aCC-BY 4.0 International license.

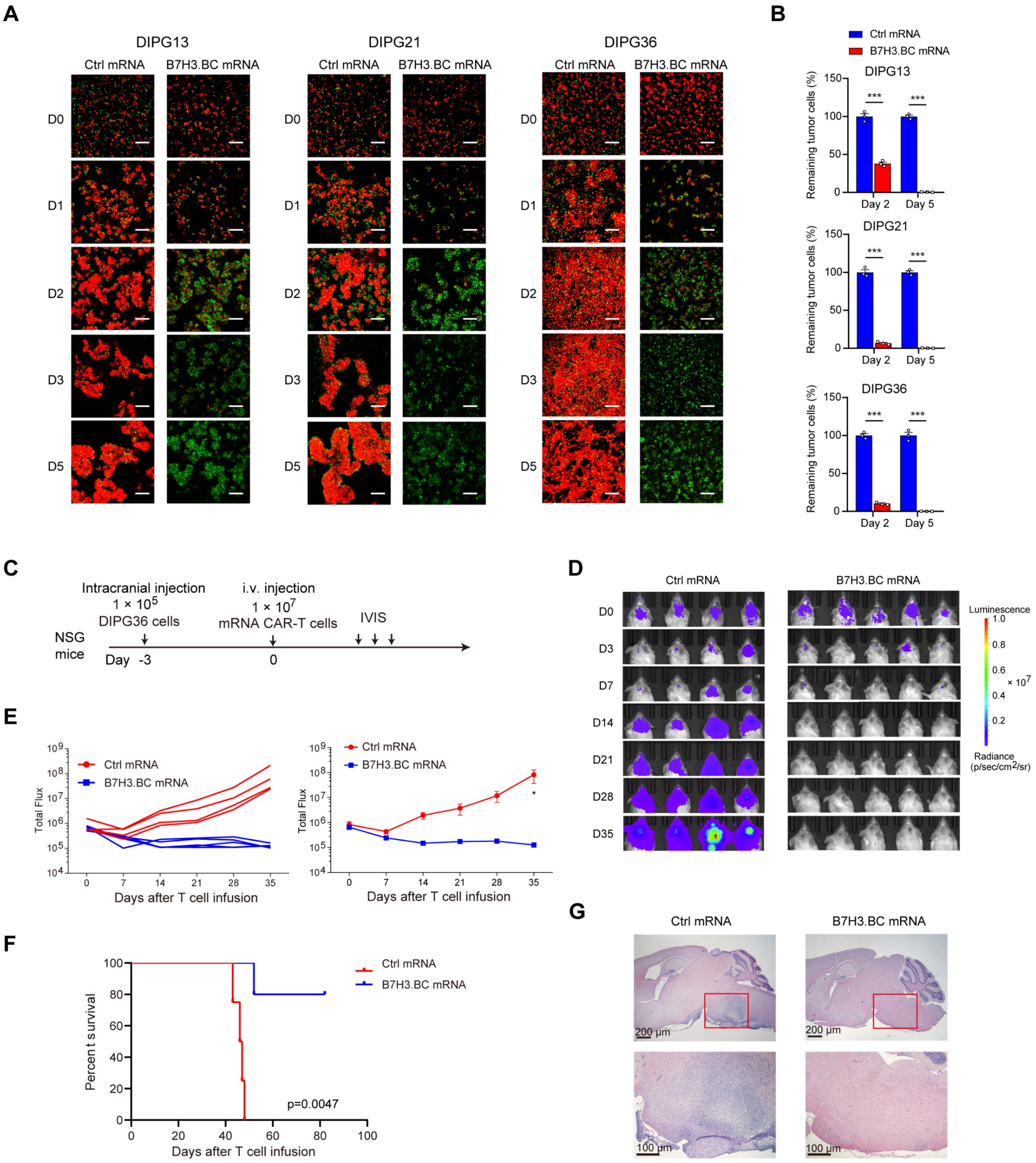

Supplementary Figure S7

bioRxiv preprint doi: https://doi.org/10.1101/2025.09.29.679095; this version posted October 1, 2025. The copyright holder for this preprint (which was not certified by peer review) is the author/funder, who has granted bioRxiv a license to display the preprint in perpetuity. It is made available under aCC-BY 4.0 International license.

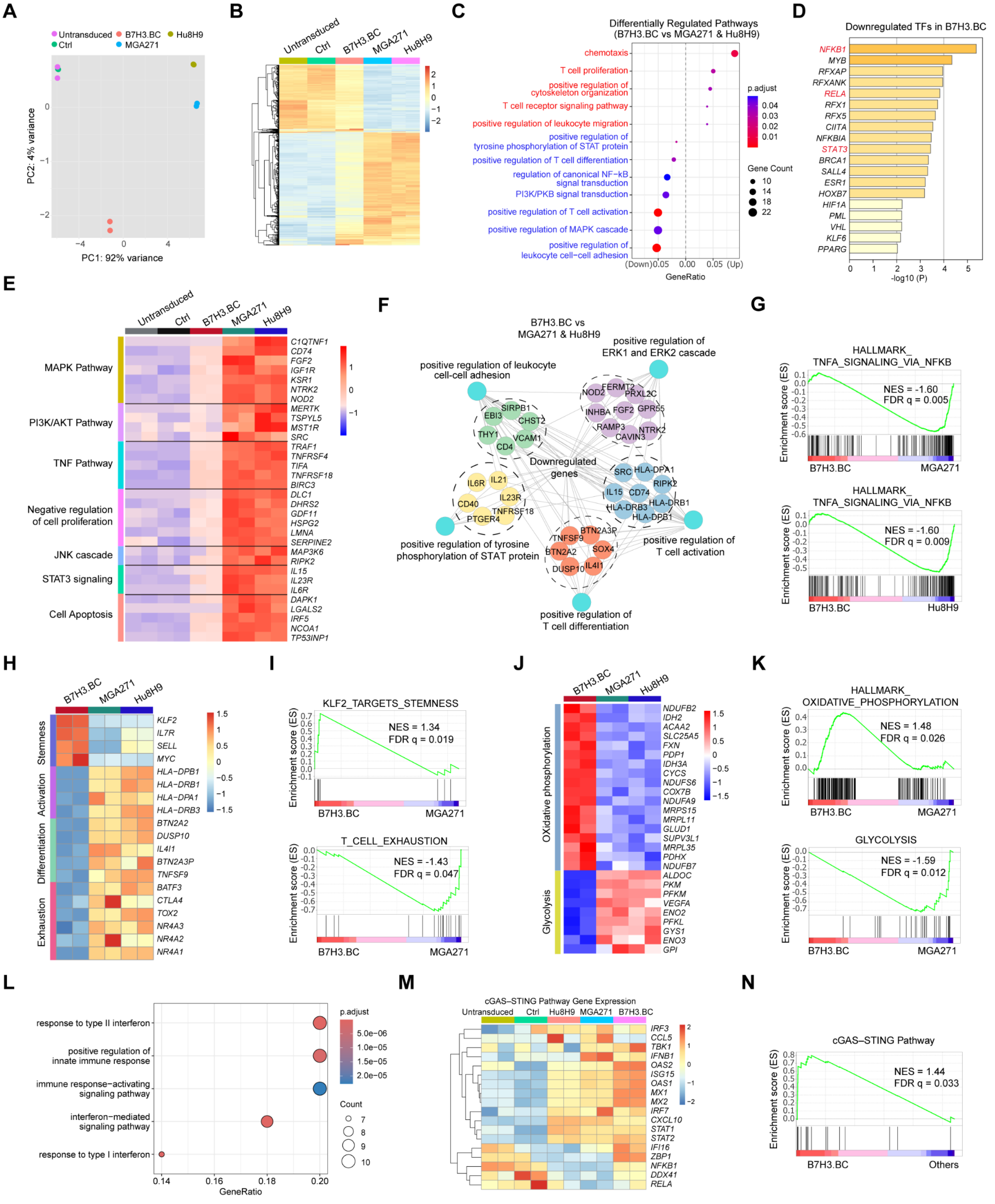

Supplementary Figure S8

bioRxiv preprint doi: https://doi.org/10.1101/2025.09.29.679095; this version posted October 1, 2025. The copyright holder for this preprint (which was not certified by peer review) is the author/funder, who has granted bioRxiv a license to display the preprint in perpetuity. It is made available under aCC-BY 4.0 International license.

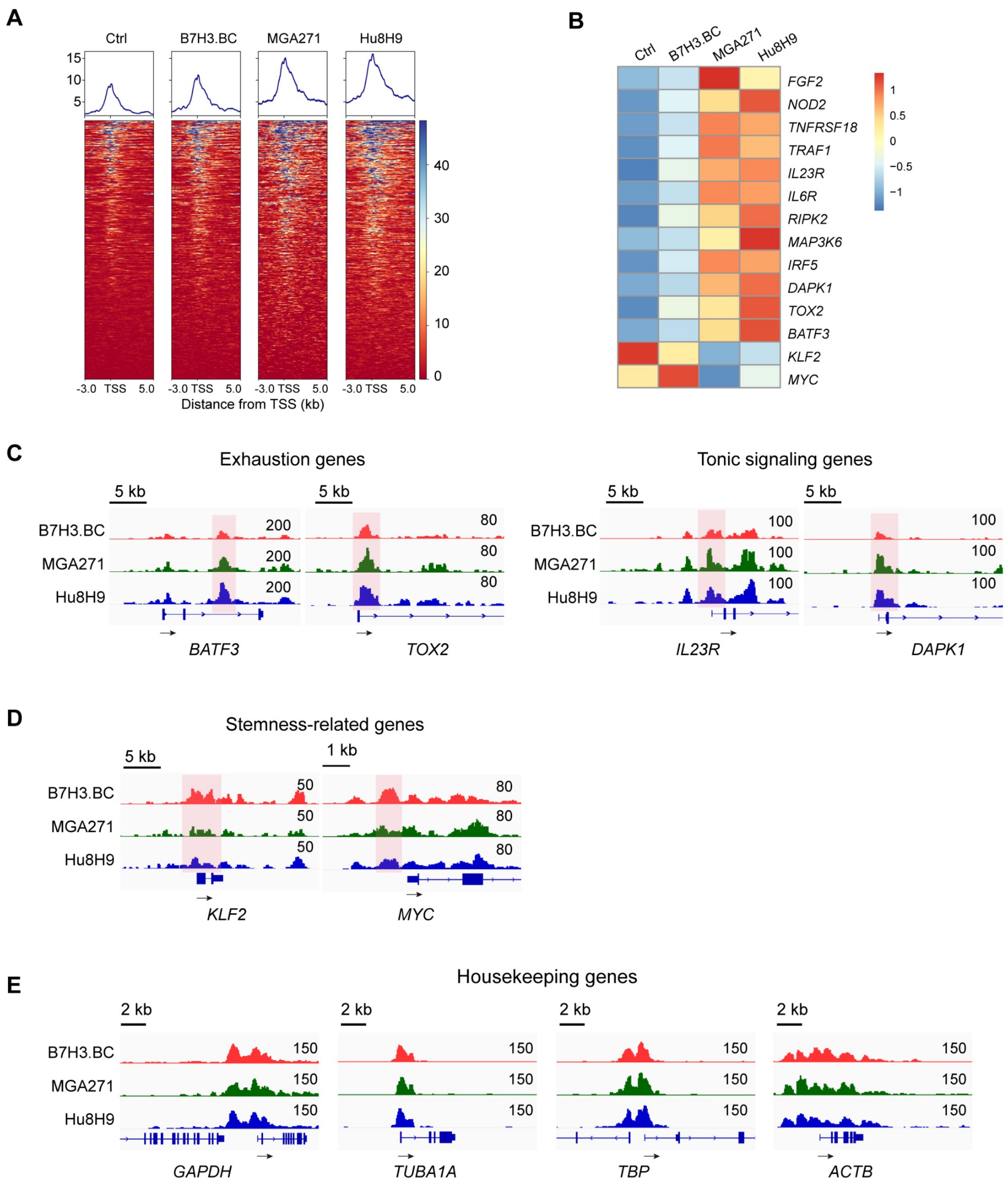

Supplementary Figure S9

bioRxiv preprint doi: https://doi.org/10.1101/2025.09.29.679095; this version posted October 1, 2025. The copyright holder for this preprint (which was not certified by peer review) is the author/funder, who has granted bioRxiv a license to display the preprint in perpetuity. It is made available under aCC-BY 4.0 International license.

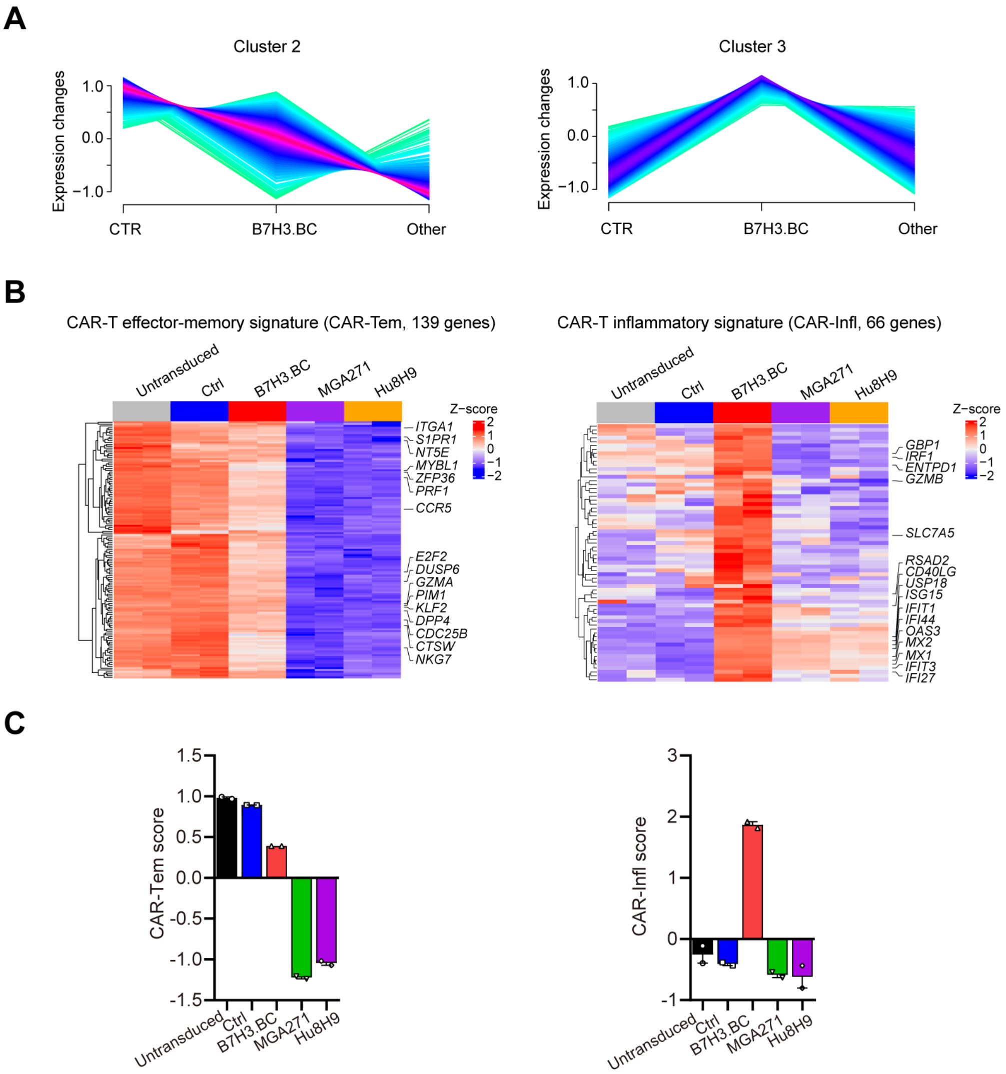

bioRxiv preprint doi: https://doi.org/10.1101/2025.09.29.679095; this version posted October 1, 2025. The copyright holder for this preprint (which was not certified by peer review) is the author/funder, who has granted bioRxiv a license to display the preprint in perpetuity. It is made available under aCC-BY 4.0 International license.

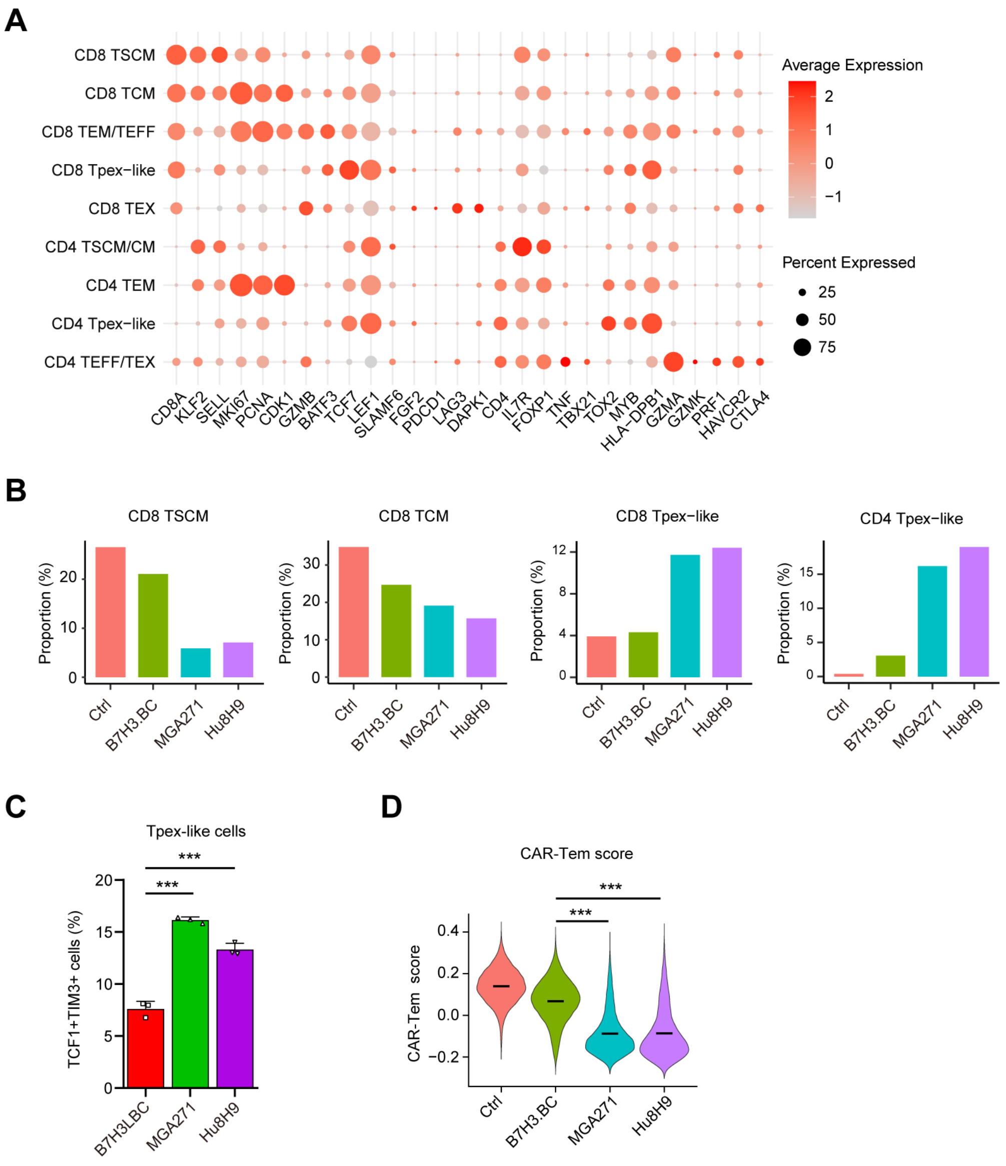

Supplementary Figure S11

bioRxiv preprint doi: https://doi.org/10.1101/2025.09.29.679095; this version posted October 1, 2025. The copyright holder for this preprint (which was not certified by peer review) is the author/funder, who has granted bioRxiv a license to display the preprint in perpetuity. It is made available under aCC-BY 4.0 International license.

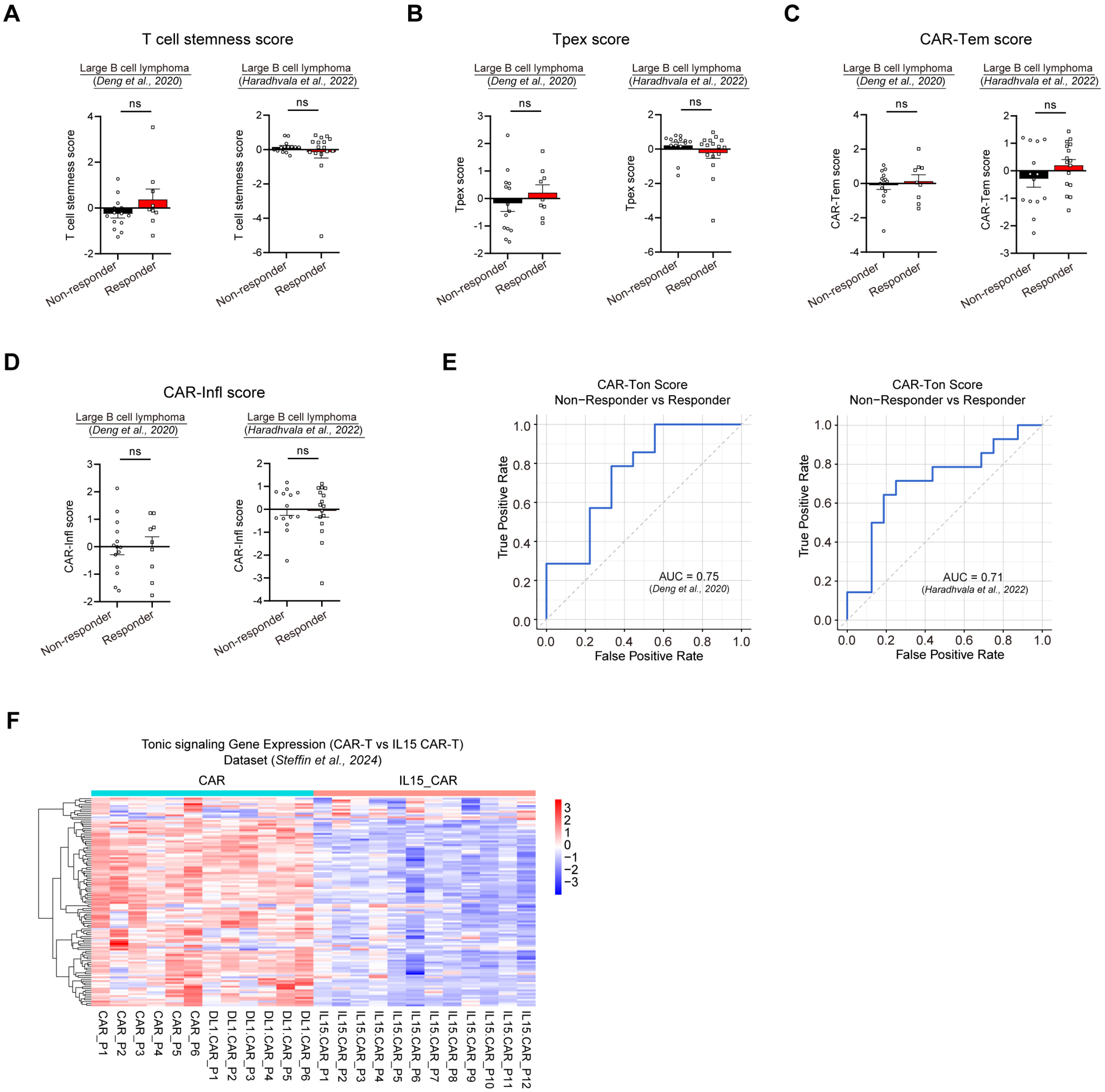

Supplementary Figure S12
